# Risk of Skin and Soft Tissue Infections among Children Found to be *Staphylococcus aureus* MRSA USA300 Carriers

**DOI:** 10.5811/westjem.2016.10.30483

**Published:** 2017-01-27

**Authors:** Lilly Cheng Immergluck, Shabnam Jain, Susan M. Ray, Robert Mayberry, Sarah Satola, Trisha Chan Parker, Keming Yuan, Anaam Mohammed, Robert C. Jerris

**Affiliations:** * Morehouse School of Medicine, Clinical Research Center, Departments of Microbiology, Biochemistry, Immunology and Pediatrics, Atlanta, Georgia; † Emory University, Department of Pediatrics, Atlanta, Georgia; ‡ Emory University, Department of Medicine, Divison of Infectious Diseases, Atlanta, Georgia; § Morehouse School of Medicine, Department of Community Health & Preventive Medicine, Atlanta, Georgia; ¶ Postgraduate Medical Institute, Anglia Ruskin University Clinical Trials Unit, Chelmsford, United Kingdom; || Pediatric Emergency Medicine Associates, Atlanta, Georgia; # Emory University, Department of Pathology, Atlanta, Georgia

## Abstract

**Introduction:**

The purpose of this study was to examine community-associated methicillin resistant *Staphylococcus aureus* (CA-MRSA) carriage and infections and determine risk factors associated specifically with MRSA USA300.

**Methods:**

We conducted a case control study in a pediatric emergency department. Nasal and axillary swabs were collected, and participants were interviewed for risk factors. The primary outcome was the proportion of *S. aureus* carriers among those presenting with and without a skin and soft tissue infection (SSTI). We further categorized *S. aureus* carriers into MRSA USA300 carriers or non-MRSA USA300 carriers.

**Results:**

We found the MRSA USA300 carriage rate was higher in children less than two years of age, those with an SSTI, children with recent antibiotic use, and those with a family history of SSTI. MRSA USA300 carriers were also more likely to have lower income compared to non-MRSA USA300 carriers and no *S. aureus* carriers. Rates of Panton-Valentine leukocidin (PVL) genes were higher in MRSA carriage isolates with an SSTI, compared to MRSA carriage isolates of patients without an SSTI. There was an association between MRSA USA300 carriage and presence of PVL in those diagnosed with an abscess.

**Conclusion:**

Children younger than two years were at highest risk for MRSA USA300 carriage. Lower income, recent antibiotic use, and previous or family history of SSTI were risk factors for MRSA USA300 carriage. There is a high association between MRSA USA300 nasal/axillary carriage and presence of PVL in those with abscesses.

## INTRODUCTION

Nationally, community-associated infections due to resistant *Staphylococcus aureus* (*S. aureus*) continue at high rates.^1^–^3^ The predominant pediatric community-associated methicillin-resistant *S. aureus* (CA-MRSA) clinical presentation remains skin and soft tissue infections (SSTI) and is seen primarily in the ambulatory setting.^4^–^7^ The prevalence of MRSA SSTI is likely under-reported in outpatient settings since many SSTIs are not submitted for culture testing. *S. aureus* infections originate from an endogenous source and, thus, carriage is a risk factor. 8,9 Most studies have evaluated MRSA carriage and its relationship to infection in hospitalized populations.^10^–^15^ Reports addressing pediatric carriage in community settings^16^–^18^,^19^,^20^ have primarily focused on carriage in the context of transmission to household contacts^21^ or known risk factors, e.g., daycare attendance^22^ or outbreak settings, e.g., newborn nurseries.^23^ There are fewer studies addressing *S. aureus* carriage among healthy children 24,25 and its association with SSTIs in these otherwise-healthy children.^6^,^26^ However, Fritz et al. demonstrated that 76% of children found to have MRSA SSTI were also colonized with MRSA.^27^ Atopic conditions, e.g., eczema, asthma, have been associated with the development of SSTIs.^28^ Atopic dermatitis is a chronic condition complicated by high rates of *S. aureus* infections, and children with this condition are known to frequently be carriers of *S. aureus*^29^

In Atlanta, Georgia the MRSA carriage rate among adults seen in the ED was 7.3%, 1,30 but the *S. aureus* carriage rate for children in Atlanta is unknown. In the U.S., the majority of CA-MRSA SSTIs have been attributed to pulsed-field type USA300,^31^,^32^ but little is known regarding what all the risk factors for CA-MRSA USA300 carriage are 3,22 or what drives this carriage to then cause SSTIs in the pediatric population. 7,33 Therefore, to explore from an epidemiological perspective how *S. aureus* carriage, and specifically MRSA USA300 carriage, is associated with development of SSTI in children, we determined carriage rates and assessed for associated risk factors among a population of children with and without a *S. aureus* SSTI in a large urban emergency department (ED). We hypothesized that MRSAUSA300 carriage was more highly associated with those who presented with SSTIs compared to those who presented without a SSTI.

## METHODS

### Study Design

This was a case control study performed in the ED of a pediatric hospital in Atlanta, Georgia. During the study period (November 2006 through April, 2008) the ED had 72,722 outpatient visits and 1,114 visits for SSTI.

### Recruitment of Study Participants

**Figure 1:**
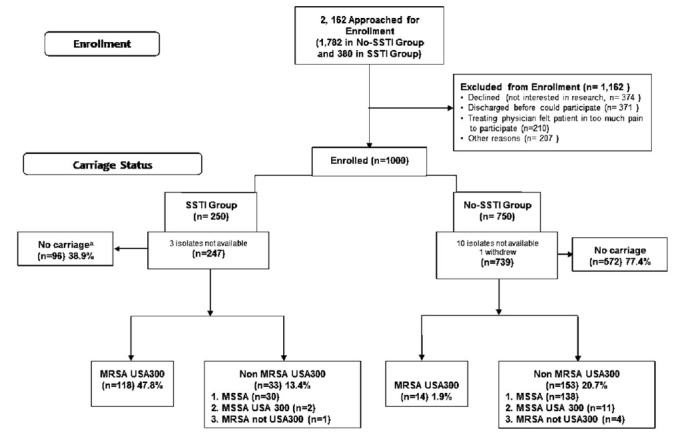
*Staphylococcus aureus* carriage enrollment flow diagram. ^a^Definition for ‘No carriage’: No detection of *S. aureus* from SSTI wound culture or no detection of *S. aureus* from cultures obtained from nasal or axillary swabs. *SSTI,* skin and soft tissue infection; *MRSA,* methicillin resistant *Staphylococcus aureus.*

Recruitment generally occurred on weekdays, 8 a.m. to midnight, and randomly selected weekend dates. (Using a random number generator, two weekend days per month were selected.) Patients younger than 21 years of age, who accessed the ED for any condition and were determined to be clinically stable by the attending physician, were eligible to participate. (Classification of “clinically stable” was based on two factors: 1) Emergency Severity Index assigned to patient (which had to be greater than or equal to three);^34^ and 2) verbal acknowledgment by the treating physician that the patient was clinically stable.) Children with and without a diagnosis of SSTI were identified by the attending physicians and were approached by study personnel until 250 children with SSTI were recruited. In selecting the 750 who lacked an SSTI, every 10^th^ patient triaged as not having SSTI and determined to be clinically stable was approached for enrollment until 750 were successfully recruited and consented. Selection of both cases and controls was concurrent (Figure 1).

### Study Procedures

After informed consent and assent (when appropriate) were obtained, participants and legal guardians were administered a survey pertaining to their demographic, personal and household members’ risk factors (Table 1). Two swabs were then collected, one each from the anterior nares and axillae. For the nares, a cotton-tipped swab (Copan Venturi Transystem® with Liquid Stuart Medium) was rotated three times in the anterior portion of each naris. For the axilla, three to five brush strokes under each axillary area were taken. Moistened swabs were then transported immediately to the clinical microbiology laboratory for plating on selective and non-selective media. The institutional review boards of participating institutions approved this study.

### Assessment of Risk Factors for CA-MRSA Carriage and Infection

We reviewed medical records of study participants for demographic information, including health insurance information, details of the treatment rendered at the relevant ED visit, and evidence of any previous hospital visits for *S. aureus* infections. In the survey, we collected information on age, race/ethnicity, gender, household income and household size. Information on past medical history was also collected using an open-ended question, “Does your child have any significant past medical history?” For those who responded “yes,” details were recorded into categories of medical conditions. Inquiry was also made about recent antibiotic use, hospitalizations, and surgeries. Participants were also asked about daycare or school attendance. We also surveyed information on household members’ use of recent antibiotics, SSTIs, hospitalizations, surgeries, dialysis, indwelling catheters, daycare attendance, and living in a closely congregated setting (jail/prison, dormitory, or military barrack) or long-term care facility within the preceding 12 months.

### Definition of *S. aureus* Carriage

We assigned *S. aureus* carriage to enrolled participants, based on evidence of *S. aureus* detection from swabs taken from nasal, or axillary areas, or specimens collected from cultured SSTIs. Because MRSA USA300 has been most tied with community-associated SSTIs, we then sub-categorized those identified as *S. aureus* carriers into “MRSA USA300 carriers” (cases) and “non-MRSA USA300 carriers” (control group 1)**.** MRSA USA300 carriers included any participant who had a MRSA isolate from nasal/axillary swabs that was typed USA300 and any participant without a positive MRSA nasal/axillary isolate who had an SSTI isolate, predictably MRSA USA300.^33^,^35^–^37^ Non-MRSA USA300 (control group 1) included all participants who had *S. aureus* isolate, not MRSA USA300 isolate, from nasal/axillary swabs and participants not found to have *S. aureus* nasal/axillary isolate but had an SSTI for methicillin-susceptible *S. aureus* (MSSA). If there was no evidence of *S. aureus* either from nasal/axillary swabs or SSTI culture, then we categorized the participant as not having *S. aureus* detected (“no *S. aureus”* carriage and assigned as control group 2).

### Characterization of *S. aureus* SSTIs

We categorized SSTIs into the following conditions: abscess (any amount of fluctuance with or without surrounding erythema), cellulitis (erythema with or without evidence of purulence), infected wound (any break in the skin integrity with surrounding erythema and/or drainage), and other.

### Laboratory Methods

#### Detection And Identification of *S. aureus* And MRSA

Swabs from enrollees were streaked within 24 hours of collection onto CHROMagar™ MRSA medium (BD Diagnostics, Sparks, MD) and Mannitol Salt Agar (MSP, Remel, Lenexa, KS). Plates were examined for typical colonies indicative of *S. aureus* and MRSA, 24 and 48 hours after inoculation and incubation at 35°C. Typical MRSA colonies on CHROMagar™ MRSA were mauve to light mauve as previously reported.^15^ *S. aureus* appeared as yellow colonies on MSP. All typical colonies were sub-cultured onto 5% sheep blood agar plates (Remel, Lenexa, KS) and tested for the presence of clumping factor and protein A (*S*taphaurex^®^, Remel, Lenexa, KS). *S. aureus* isolates were frozen at −80°C until molecular characterization was performed.

#### Antibiotic Susceptibility Testing

Antimicrobial susceptibility testing was performed using MicroScan (Siemens Healthcare, Deerfield, IL). We made interpretations according to breakpoints established by the National Committee for Clinical Laboratory Standards Institute.^38^ Antibiotics tested included penicillin, oxacillin, erythromycin, clindamycin, linezolid, trimethoprim-sulfamethoxazole, ciprofloxacin, quinupristin-dalfopristin, tetracycline, gentamicin and rifampin.

#### Pulsed-Field Gel Electrophoresis (PFGE) Typing

We performed strain typing by PFGE with the *Sma*I restriction enzyme as previously described,^39^ using *Salmonella enterica* serovar Braenderup H9182 as the normalization standard. Gel images were compared using BioNumerics version 5.01 software (Applied Maths, Austin, TX) and assigned to previously defined pulsed-field types 39,40 at 95% relatedness by use of Dice coefficients and the unweighted-pair group method using average linkages.^41^

#### Staphylococcal Chromosome Cassette mec (SCCmec) Typing

Among MRSA carriage isolates, identification of the SCC*mec* element was performed by polymerase chain reaction (PCR) analysis designed to identify SCC*mec* types II and IV only as previously reported.^37^ *SCCmec* IV bearing strains were further sub-typed with primers for SCC*mec* IVa as described.^42^

#### Panton Valentine Leukocidin (PVL) Testing

We used PCR to identify the genes encoding *LukS-PV* and *LukF-*PV as reported by Lina et al.^43^

### Statistical Analysis

We used descriptive statistics to provide mean value and relative frequency of each variable for all study participants and then for subgroups based on definitions of *S. aureus* carriage and the presence or absence of *S. aureus* SSTI at the time of enrollment. The relationships between MRSA USA300 and non-MRSA USA300 (MSSA USA300, MSSA not USA300, and MRSA not USA300) and presence or absence of SSTI, along with epidemiological risk factors were investigated by chi-square and t-test statistics as appropriate. We performed sensitivity analyses on risk factors to compare MRSA USA300 cases to two different control groups as described above. Certain variables were re-coded to fewer categories in order to conduct statistical analysis. For example, we grouped annual household incomes into three categories: low (< $20,000), moderate (≥$20,000 and ≤$75,000), or high (>$75,000). Household income and household size were also combined to factor in household income based on household size. We divided the midpoint of the household income reported by the household size. To look for associations between SSTI and atopic conditions, the past medical conditions were categorized into “atopic conditions” (eczema, allergies, asthma) or “not atopic conditions.” We applied logistic regression to assess the bivariate association between carriage status and the presence of risk factors. Bivariate logistic regression analysis was also applied for those factors *a priori* thought to be associated with risk of MRSA USA300 and then multivariate logistic regression analysis was performed to assess the association between MRSA USA300 and non MRSA USA300 carriage status adjusted for those risk factors. Similarly, we performed multivariate logistic regression analysis to assess MRSA USA300 and no *S. aureus* carriage, adjusted for risk factors determined *a priori*. We used the log likelihood ratio test to assess the significance of variables on the odds of *S. aureus* carriage and, specifically, MRSA USA300 carriage. Likewise, we calculated odds ratios as estimates of relative risks, indicating the magnitude of associations, along with corresponding 95% confidence intervals (CI). All tests for significance were two-tailed, and a p-value of ≤ 0.05 was considered significant. We performed statistical analysis using SAS 9.1(SAS Institute, Cary, NC). Survey questionnaire responses were based on self-reports, which were administered as personal interviews conducted in the privacy of the ED examination room.

## RESULTS

### Study Population Characteristics

From November 2006 through April 2008, 2,162 children were approached in the pediatric ED for enrollment. Sixty-six percent (250/380) of children with an SSTI and 42% (750/1,782) of children who lacked an SSTI agreed to participate (Figure 1). Reasons for declining enrollment were similar in both groups.

### Characteristics of *S. aureus* Carriers

#### Risk factors for *S. aureus* Carriage

Participants identified as MRSA USA300 carriers compared to non-MRSA USA300 (control group 1) were less than two years of age, presented with or had previous SSTI, had recent antibiotic use, and had a household member with past SSTI (Table 1).

In comparison, non-MRSA USA300 carriers, who were mostly MSSA carriers, were more likely to have a household member employed in healthcare field, have an atopic condition, and if they were less than or equal to two years of age, attended daycare. When we compared MRSA USA300 to those who had no evidence of any *S. aureus* carriage (control group 2), we determined that receiving public health insurance and having lower income also were significant risk factors.

Table 2 shows the adjusted odds ratio for the epidemiological risk factors associated with MRSA USA300 carriers compared to non-MRSA USA300 carriers and to those with no evidence of *S. aureus* carriage. In these multivariate analyses, we observed that those younger than two years of age who attended daycare were almost four times more likely to be MRSA USA300 carriers (aOR 3.67, 95% CI 1.07–12.57) compared to non-MRSA USA300 carriers. Similarly, MRSA USA300 carriers had an adjusted odds ratio of 2.51(95% CI 1.47–29) compared to non-MRSA USA300 carriers for recent antibiotic use, 4.88 (95% CI 2.08–11.43) for past history before current episode of SSTI and 3.91 (95% CI 1.76–8.69) for family history of SSTI. These adjusted odds remained similarly higher for MRSA USA300 carriers compared to those who were not found to have any evidence of *S. aureus* carriage for all the risks except daycare attendance in those younger than two years of age. MRSA USA300 carriers were also 4.18 (aOR, 95% CI 1.57–11.12) and 3.13 (aOR 95% CI 1.37–7.16) more likely to have an income <$20,000 compared to non-MRSA USA300 carriers or those with “no *S. aureus”* carriage, respectively.

Among those with an SSTI, 48% (118/247) were MRSA USA300 carriers compared to 13% (33/247) non-MRSA USA300. In contrast, among those without SSTI at enrollment, only 2% (14/739) were MRSA USA300 carriers compared to 21% (153/739) non-MRSA USA300 (Table 3).

**Figure 2:**
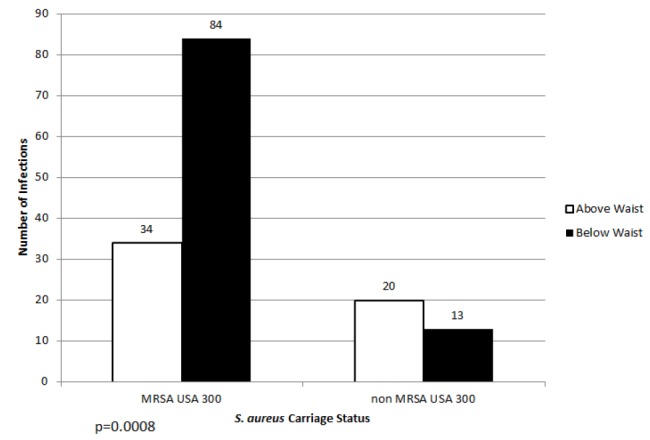
*S. aureus* carriage strains and body locations of skin and soft tissue infections. *SSTI,* skin and soft tissue infection; *MRSA,* methicillin resistant *Staphylococcus aureus*.

No MRSA carriers were found among those who had SSTI cultures that yielded no growth (14) or *S. pyogenes.*^4^ MRSA USA300 carriers (71.2%, 84/118) were also more likely than non-MRSA USA300 carriers (39.4%, 13/33) to have an SSTI located below the waist than above the waist (p=0.0008) (Figure 2).

#### *S. aureus* Carriage Rates Based on Nasal and Axilla Cultures

**Figure 3:**
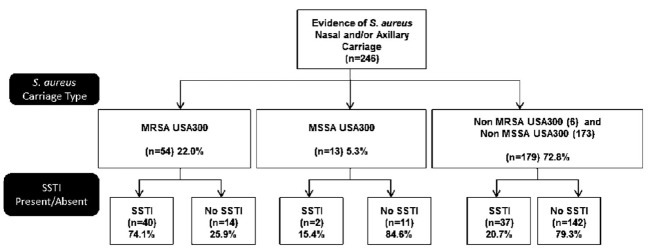
Distribution of nasal and axillary *S. aureus* carriage types between SSTI and no-SSTI groups. Note: No *S. aureus* carriage was detected in 668 swabs taken from either nasal, axillary areas: 96/668 were from SSTI group and 572/668 were from No SSTI group. *SSTI,* skin and soft tissue infection; *MRSA,* methicillin resistant *Staphylococcus aureus*.

The positivity rate was 25% (246/986) for *S. aureus* based only from nasal or axilla cultures. Of those with *S. aureus*, the carriage rates for MRSA USA300 and MSSA USA300 were 22.0% (54/246) and 5.3% (13/246), respectively; the remaining non-USA300 were mostly all MSSA (70.3%, 173/246) and very few MRSA (2.4%, 6/246) (Fig. 3). Significant risk factors for nasal/axillary MRSA USA300 carriage were the same as stated previously (data not shown).

### Concordance between Nasal and Axillary *S. aureus* Carriage Isolates

Among 237 with positive *S. aureus* nasal isolates, 183 (77.25%) had positive *S. aureus* axillary isolates. Conversely, 183 of the 192 (95.3%) axillary carriers were also nasal carriers. Among the 57 *S. aureus* nasal and axillary pairs designated for typing, there was concordance of PFGE types in 53 pairs (93.3%). We found discordant pulsed-field types for three MSSA carriers who lacked an SSTI, of which two were associated with USA300. There was discordant pulsed-field typing of one MRSA carrier with an SSTI also associated with USA300.

#### Microbiological And Molecular Profiles of *S. aureus* Nasal and Axillary Carriage And SSTI Isolates

Susceptibility to ciprofloxacin, clindamycin, erythromycin, gentamicin, linezolid, rifampin, trimethoprim-sulfamethoxazole, tetracycline, and vancomycin was shared between nasal/axillary carrier isolates and the associated SSTI isolates in 83.3% (10/12) MSSA isolates, and 93.3% (28/30) MRSA isolates.

There were 302 *S. aureus* nasal/axillary carriage isolates from 246 participants available for molecular testing. USA300 accounted for 88.3% (53/60) of all MRSA isolates. There was a significant difference between rates of MRSA USA300 nasal/axillary carriage among those with an SSTI (92.9%, 39/42) and those who lacked an SSTI (77.8%, 14/18, p=0.05). All nasal/axillary MRSA USA300 isolates had a SCC*mec* type IV element and 74.5% (41/55) were SCC*mec* type IVa. The PVL genes were found in 67% (12/18) of these MRSA carriage isolates from patients who lacked an SSTI and in 92.8% (39/42) of MRSA carriage isolates with an SSTI (p=0.009); all 39 PVL + MRSA carrier isolates were USA300.

## DISCUSSION

In our study we hypothesized that children found to have SSTIs are more likely to be MRSA carriers and, in particular, MRSA USA300 carriers compared to children who presented without SSTI. We found that children younger than two years were 3.67 [95% CI, 1.07–12.57] times more likely to be MRSA USA300 carriers than all other *S. aureus* PFGE types; this observation persisted even after adjusting for factors such as daycare (Table 2). Most likely this is related to the naturally higher bacterial load and moist environment of the diapered area.^44^ MRSA USA300 carriers were also more likely than all other *S. aureus* carriers to have SSTIs below the waist, even though the overall distribution of SSTI types was similar between MSSA and MRSA carriers (Figure 2). These two findings were consistent with what has been reported by Fritz et al.^27^ Our risk factors for MRSA USA300 carriage in those children under two years, e.g., recent antibiotic use, history of SSTI, are similar to what others have reported for CA-MRSA infections where there was no pulsed-field typing done (Table 2). Our proxies for lower socioeconomic factors (low income, <$20,000, public health insurance) were more likely among those found to be MRSA USA300 carriers, which may be tied in with why household crowding is a risk for CA-MRSA infections (Table 2).

We did not find daycare or school attendance as a risk for SSTI among those who were MRSA USA300 carriers, and in fact among those who presented with SSTI, non-MRSA USA300 carriers had higher rates of daycare or school attendance than MRSA USA300 carriers (Table 2). Although daycare has been cited as a risk factor for CA-MRSA infections in some studies,^45^ our finding is consistent with what others have also reported.^44^ We postulate this may be attributed to the fact that daycare or school promotes close contact of children, and thus spread of infections among all types of *S. aureus* carriers, not just MRSA USA300. Others have suggested that daycare attendees may have more frequent changes of diaper and, consequently, less time where skin is directly exposed to stool or moisture.^44^

We found a history of atopic conditions to be associated with non-MRSA USA300 carriers with an SSTI, which was not found among MRSA USA300 carriers (Table 2). Interestingly, having a history of atopic conditions (including eczema or atopic dermatitis) did not occur more frequently among those who presented with an SSTI, even though this condition is clearly associated with compromised skin integrity and children with atopic dermatitis are known to have high carriage rates of *S. aureus.* 46,47 Little has been published on CA-MRSA carriage and its association to infections among those with atopic dermatitis,^48^ despite the fact that these patients have a predisposition for being heavily colonized or infected with *S. aureus.*^49^–^51^ In the study published by Matiz, et al., they also did not find higher rates of CA-MRSA among their atopic dermatitis population.^52^ This observation may be due in part to a “protective effect” afforded by presence of other non- CA-MRSA organisms, and other factors, e.g., skin levels of fibronectin, fibrinogen.^53^

We found that even though the rate of household members with an SSTI was higher among MRSA USA300 carriers, almost 10% of non-MRSA USA300 carriers who presented without an SSTI had a household member with a previous SSTI (Table 2); this observation further demonstrates how widespread *S. aureus*-related SSTIs are in the community. In other studies, more than 50% of household contacts of patients with *S. aureus* infections have been shown to be *S. aureus* carriers.^27^ The specific factors that lead a household member with *S. aureus* carriage to a household member with an infection may be multifactorial: the specific strain (e.g., specific virulence factors produced by USA300); host immunity (e.g., skin integrity); or environmental conditions (e.g., household crowding, extent of sharing of household items that contact the skin).

Not surprisingly, we also found that MRSA USA300 carriage was at least 10 fold higher in children with SSTI compared to those who lacked an SSTI (Table 3). In contrast, the no- MRSA USA300 carriage rates (which were mostly MSSA non-USA300 strains) were similar between SSTI and no SSTI, suggesting that MSSA carriage is not predictive of development of an SSTI. Our MRSA carriage rate was lower than the 61% observed among those with SSTIs reported by Fritz et al.^27^ However, in our study, we also addressed the impact of specific strain types, namely the impact of MRSA USA300 carriage. There was also more heterogeneity in pulsed-field types among MRSA carriers who lacked an SSTI compared to those with an SSTI. This also suggests that MRSA USA300 carriage is predictive of development of a MRSA SSTI, particularly of abscesses large enough to warrant the clinician’s decision to culture.

Our study supports the notion that PVL cytotoxin in MRSA USA300 carriage isolates may be a contributing factor to the development of an abscess type of SSTI as has been suggested by others.^1^,^33^ The PVL genes were found in all of the MRSA USA300 nasal and axillary carriage isolates. Further study is needed to understand what additional virulent factors are tied to MRSA USA300 carriage and specifically which virulence factors contribute most to the development of recurrent SSTIs or more invasive infections.

The discordance between nasal and axillary isolates was higher for MSSA than MRSA and more often seen in MSSA carriers who did not present with an SSTI. We also found that MSSA carriers were more likely to have discordance with their SSTI isolates, which were found to be MRSA. These discordances taken together with the observation that there was no predictive association seen with MSSA carriage and development of an SSTI further support the notion that specific strains among MSSA, more so than MRSA, were likely to carry genetic backgrounds that were not disease producing.^26^

The MRSA nasal/axillary carriage rate among patients with an SSTI was far less than the MRSA SSTI rate among all cultured SSTIs. It is possible that many of those not found to be *S. aureus* carriers but had *S. aureus* SSTIs might be transient carriers, who simply were not detected at the time of enrollment in our point prevalence study. Intrinsic factors related to specific clonal types may be responsible and explain why some strains have the propensity for persistent carriage and subsequent development of an SSTI while others do not.

## LIMITATIONS

This study was limited since it was a convenience sample, and thus, a point-prevalence determination of MRSA nasal and axillary carriage on the day patients were enrolled. We were not able to assess for differences between transient *S. aureus* carriers and persistent carriers. *S. aureus* isolates obtained from the SSTI cultures were also not available to perform pulsed-field typing or other molecular testing. Based on the fact that the nares have been considered to be the most frequent site for *S. aureus* carriage and a risk factor for subsequent staphylococcal infection, 19,54,55 our study collected from this area to determine carriage rates. It is possible that this site may not yield the highest possible number of *S. aureus* carriers^56^ and therefore is an underestimation of the true *S. aureus* prevalence carriage rate in our population. Miller et al. found they would have missed 48% of *S. aureus* carriage by conducting a nares-only surveillance. (They screened for carriage from three sites: nares, oropharynx, and inguinal.)^26^ However, our *S. aureus* nasal carriage rates are similar to what others have reported in otherwise-healthy children.^6^ Future studies may need to include broth-based cultures of specimens obtained from nasal, oral pharyngeal, and inguinal area so as to capture the highest number of *S. aureus* carriers.^57^ Culturing additional body sites may also shed more information as to why some have reported USA300 strains predominantly colonizing groin areas compared to non-USA300 strains, which were found more frequently in the oropharynx.^58^ We also recognize that the data were collected during the height of the CA-MRSA epidemic in this country; however, we believe that based on more recent studies (Immergluck L, personal communication on unpublished data of 85 children with SSTI enrolled from the same hospitals) SSTIs due to CA-MRSA remain a constant infection in our ambulatory and ED settings. Understanding the epidemiology, particularly as it relates to the specific circulating strains and the antibiotic profile (phenotype) of these strains that cause infections, is critical as we continue to revise the treatment guidelines for empiric treatment and for outlining when routine culture should be done in these settings. Moreover, the MRSA strains associated with carriage are also important to delineate, given the association between carriage and infection.

## CONCLUSION

We found children younger than two years were at highest risk for MRSA USA300 carriage. We also found lower income, recent antibiotic use, previous or family history of SSTI (but not daycare) to be risk factors for MRSA USA300 carriage. There is clearly a high association between MRSA USA300 nasal/axillary carriage and presence of PVL in those found to have the specific SSTI diagnosis of abscesses. Our study pulsed-field typed the wide array of both MRSA and MSSA non-USA300 carriage isolates among children with no SSTI infections. The propensity for MRSA USA300 infections to occur in the groin and buttock areas is likely related to higher bacterial burden provided through the moist milieu in this area. Our finding of higher MRSA USA300 carriage in children younger than two years with SSTIs needs to be further explored. Additional studies are also needed to define what host and what specific pathogenic factors might distinguish those who become infected to continue to become persistent MRSA USA300 carriers from those who are merely transient MRSA carriers. Given the strain diversity for both MRSA and MSSA and the variability in which strains spread among household members, more studies are needed to help understand the virulence and host factors that allow certain strains to move from carriage to primary and recurrent infections if we are to wage a successful battle to decrease SSTI in this population.

## Figures and Tables

**Table 1:** Descriptive population characteristics of patients in study of risk of skin and soft tissue infections in children who are MRSA carriers.

Characteristic variable	Cases MRSA USA300 n=132(%)	Control 1 non-MRSA USA300** n=186(%)	P value	Control 2 No *S. aureus* n=572(%)	P value
Demographic information of participants					
Gender			0.0685		0.0747
Male	63 (47.7)	108 (58.1)		322 (56.3)	
Female	69 (52.3)	78 (41.9)		250 (43.7)	
Age distribution*			< 0.0001		0.0009
Birth through 2 years	60 (45.5)	29 (15.6)		162 (28.3)	
>2 through 5 years	28 (21.2)	21 (11.3)		139 (24.3)	
>5 through 8 years	9 (6.8)	42 (22.6)		99 (17.3)	
>8 through 12 years	15 (11.4)	51 (27.4)		81 (14.2)	
>12 years	20 (15.1)	43 (23.1)		91 (15.9)	
Family size			0.9164		0.584
0–4	78 (59.1)	111 (59.7)		323 (56.5)	
> 4	54 (40.9)	75 (40.3)		249 (43.5)	
Race/ethnicity			0.4775		0.0777
White	38 (28.9)	44 (23.7)		121 (21.2)	
Black	91 (68.9)	135 (72.5)		421 (73.6)	
Other	3 (2.3)	7 (3.8)		30 (5.2)	
Insurance type			0.1111		0.0158
Self pay	11 (8.3)	25 (13.8)		79 (13.8)	
Private	32 (24.3)	54 (29.8)		183 (32.0)	
Public	89 (67.4)	102 (56.4)		306 (53.5)	
Not reported				4 (0.7)	
Annual household income			0.0079		0.0027
Not reported	31 (23.5)	32 (17.2)		126 (22.0)	
<$20,000	80 (60.6)	98 (52.7)		264 (46.2)	
$20,00–$75,000	13 (9.9)	21 (11.3)		105 (18.3)	
>$75,000	8 (6.0)	35 (18.8)		77 (13.5)	
Participant risk factors					
Presence of SSTI			<0.0001		<0.0001
No	14 (10.6)	153 (82.3)		572 (100)	
Yes	118 (89.4)	33 (17.7)		0 (0.0)	
Prior atopic condition ***			0.0062		0.5466
No	114 (86.4)	137 (73.7)		482 (84.3)	
Yes	19 (13.6)	49 (26.3)		90 (15.7)	
Recent hospitalization or surgery			0.9416		0.1127
No	109 (82.6)	153 (82.3)		502 (87.8)	
Yes	23 (17.4)	33 (17.7)		70 (12.2)	
Yes	7 (5.3)	13 (7.0)		24 (4.2)	
Employed in healthcare field			0.0131		0.3174
No	109 (82.6)	131 (70.4)		450 (78.7)	
Yes	23 (17.4)	55 (29.6)		122 (21.3)	
History of residing in congregate setting			0.111		0.0728
No	130 (98.5)	177 (95.2)		543 (94.9)	
Yes	2 (1.5)	9 (4.8)		29 (5.1)	

* For the multivariate analyses, the age groups were re categorized into 3 groups (birth through 2 years, >2 through 5 years, and >5 years) and Control Group 1, p=0.9129, and for Control Group 2, p=0.0359.

** This analyses was re-run excluding those which were determined to be MSSA USA300 (n=12) from the *S. aureus* non-MRSA USA300 cohort, and the significance levels (p<0.05) for the risk factors remained unchanged.

*** Prior atopic condition: eczema, allergies, and asthma.

*SSTI,* skin and soft tissue infection; *MRSA,* methicillin resistant *Staphylococcus aureus*.

**Table 2:** Multivariate logistic regression analysis of risk factors associated with MRSA USA300, Non-MRSA USA300 carriage, and no *S. aureus* carriage.

Risk factor	Odds ratio (non MRSA USA300)	95% CI (non MRSA USA 300)	Odds ratio (no *S. aureus*)	95% CI (no *S. aureus*)
Interaction between age and daycare				NS
>2 through 5 years	1.00			
Birth through 2 years	3.67	1.07–12.57		
>5 years	1.00		1.00	
Birth through 2 years	11.47	4.33–30.42	2.14	1.32–3.48
>2 years through 5 years	3.13	1.29–7.56	1.02	0.58–1.79
Interaction between age and no daycare				NS
>2 through 5 years	1.00			
Birth through 2 years	0.78	0.23–2.67		
>5 years	1.00			NS
Birth through 2 years	1.13	0.23–5.52		
>2years through 5 years	1.45	0.19–11.03		
Income				
>$75,000	1.00		1.00	
Not reported	3.21	1.09–9.49	2.13	0.87–5.21
<$20,000	4.18	1.57–11.12	3.13	1.37–7.16
$20,000–$75,000	3.54	1.06–11.82	1.37	0.51–3.68
Prior atopic condition *				NS
Yes	1.00			
No	2.47	1.19–5.12		
Recent antibiotic use				
No	1.00		1.00	
Yes	2.51	1.47–2.90	2.42	1.58–3.71
Past history of SSTI				
No	1.00		1.00	
Yes	4.88	2.08–11.43	4.45	2.46–8.05
Family history of SSTI				
No	1.00		1.00	
Yes	3.91	1.76–8.69	3.42	2.06–5.67

Multivariate risk analyses compared cases (MRSA USA300) to the two different controls, non MRSA USA300 (n=186), and no *S. aureus* (n=572).

* Prior atopic condition: eczema, allergies, and asthma.

*SSTI,* skin and soft tissue infection; *MRSA,* methicillin resistant *Staphylococcus aureus.*

**Table 3:** Relationship of *S. aureus* carriage and presence of skin and soft tissue infections.

Carriage status	SSTI, n= 247 (%)	No SSTI, n=739 (%)	Odds ratio (95%, CI)	P-value
No *S. aureus* (n=668)	96 (38.9)	572 (77.4)	1.0	
MRSA USA300 (n=132)	118 (47.7)	14 (1.9)	50.21 (27.71–91.01)	<0.0001
Non MRSA USA300 (n=186)	33 (13.4)	153 (20.7)	1.29 (0.83–1.98)	0.26

*SSTI,* skin and soft tissue infection; *MRSA,* methicillin resistant *Staphylococcus aureus*.
